# The p38^MAPK^-MK2 Signaling Axis as a Critical Link Between Inflammation and Synaptic Transmission

**DOI:** 10.3389/fcell.2021.635636

**Published:** 2021-01-28

**Authors:** Edward Beamer, Sonia A. L. Corrêa

**Affiliations:** Faculty of Science and Engineering, Department of Life Sciences, Manchester Metropolitan University Manchester, Manchester, United Kingdom

**Keywords:** MK2, p38^MAPK^, synaptic plasticity, mGluR-LTD, neuroinflammation, cognition, hippocampus, AMPAR trafficking

## Abstract

p38 is a mitogen-activated protein kinase (MAPK), that responds primarily to stress stimuli. p38 has a number of targets for phosphorylation, including MAPK-activated protein kinase 2 (MK2). MK2 primarily functions as a master regulator of RNA-binding proteins, indirectly controlling gene expression at the level of translation. The role of MK2 in regulating the synthesis of pro-inflammatory cytokines downstream of inflammation and cellular stress is well-described. A significant amount of evidence, however, now points to a role for the p38^MAPK^-MK2 signaling axis in mediating synaptic plasticity through control of AMPA receptor trafficking and the morphology of dendritic spines. These processes are mediated through control of cytoskeletal dynamics via the activation of cofilin-1 and possibly control of the expression of Arc/Arg3.1. There is evidence that MK2 is necessary for group I metabotropic glutamate receptors long-term depression (mGluR-LTD). Disruption of this signaling may play an important role in mediating cognitive dysfunction in neurological disorders such as fragile X syndrome and Alzheimer’s disease. To date, the role of neuronal MK2 mediating synaptic plasticity in response to inflammatory stimuli has not yet been investigated. In immune cells, it is clear that MK2 is phosphorylated following activation of a broad range of cell surface receptors for cytokines and other inflammatory mediators. We propose that neuronal MK2 may be an important player in the link between inflammatory states and dysregulation of synaptic plasticity underlying cognitive functions. Finally, we discuss the potential of the p38^MAPK^-MK2 signaling axis as target for therapeutic intervention in a number of neurological disorders.

## Introduction

Over recent years, the importance of bidirectional cross-talk between the immune system and central nervous system has become increasingly clear. Immune responses are subject to neuroendocrine modulation (Taub, 2008), while inflammatory signals mediate the activity of neural networks (Schneider et al., 1998; Pickering et al., 2005; Clarkson et al., 2017). Molecules that were previously defined by their contribution to the functioning of the immune system, such as tumor necrosis factor α (TNFα), have been shown also to play important roles in regulating neuronal activity (Shatz, 2009). Conversely, a role in immune function has emerged for molecules, such as γ-Aminobutyric acid (GABA), defined by their role in neurotransmission (Kerage et al., 2019). The importance of an inflammatory contribution to neurological disorders has become increasingly clear and mechanisms through which inflammatory signaling is transduced into neuronal responses are of particular interest. The p38 mitogen-activated protein kinase (MAPK, p38^MAPK^), phosphorylates MAPK-activated protein kinase 2 (MK2). The role of this p38^MAPK^-MK2 signaling axis in cellular responses to stress and inflammatory signals, including control of the synthesis and release of inflammatory signaling molecules, has been delineated in detail, largely from immune cells (Gaestel, 2006; Menon and Gaestel, 2018). In neurons, however, the p38^MAPK^-MK2 signaling axis is responsible for mediating neurotransmission in response to activation of perisynaptic group I metabotropic glutamate receptors (mGluR1/5) on dendritic spines. Expressed in neurons throughout the brain, activated in response to inflammatory cues and with a demonstrated role in mediating synaptic plasticity, the p38^MAPK^-MK2 signaling axis is an attractive candidate for mediating crosstalk between inflammatory and neuronal signaling. Here, we will discuss what is known about activation of the p38^MAPK^-MK2 axis in response to inflammatory stimuli, the role it plays in mediating synaptic plasticity, discuss the potential of this signaling pathway for mediating cross-talk between inflammatory signals and neurotransmission and discuss settings within which targeting this signaling system may prove efficacious.

## The p38^*MAPK*^-MK2 Signaling Axis

MAPK-signaling cascades are highly conserved intracellular signaling pathways that convert external stimuli, usually through cell-surface receptors, into a range of cellular responses (Seger and Krebs, 1995). These signaling pathways are ubiquitous across the eukaryotic domain (Avruch, 2007) and mediate a great diversity of cellular processes, such as proliferation, differentiation, or apoptosis (Qi and Elion, 2005; Keshet and Seger, 2010; Morrison, 2012). These processes largely control the expression of a network of regulatory genes (Whitmarsh, 2007). Each cascade of conventional MAPKs is composed of a set of three sequentially acting kinases: an MAPK, an MAPK kinase (MAP2K), and an MAP2K kinase (MAP3K) (Cargnello and Roux, 2011). The MAP3Ks, at the top of the cascade, are protein serine/threonine kinases, often activated through phosphorylation and/or as a result of their interaction with a small GTP-binding protein of the Ras/Rho family in response to extracellular stimuli (Chiariello et al., 2010). MAP3K activation leads to the phosphorylation and activation of a MAP2K, which then activates a MAPK through dual phosphorylation on threonine and tyrosine residues within a conserved threonine-X-tyrosine motif (Cargnello and Roux, 2011).

p38^MAPK^ is a MAPK, that in mammalian cells is expressed in four splice variants: p38α, p38β, p38γ, and p38δ, with differential phosphorylation of molecular targets (Cuadrado and Nebreda, 2010). P38^MAPK^ is activated by the MAP2Ks, MKK3, and MKK6 (Zarubin and Han, 2005), via dual phosphorylation on threonine-180 and tyrosine-182 (Raingeaud et al., 1995). The principal role of p38^MAPK^ is in co-ordinating molecular responses within the cell to stimuli associated with diverse stressors (Coulthard et al., 2009). Stressors can involve changes in the immediate extracellular microenvironment, such as osmotic (Westfall et al., 2004) or thermal (Li et al., 2018) stress, or the detection of pathogens (Cargnello and Roux, 2011). Alternatively, pathology at the level of organ or organism can be detected through intercellular signaling molecules, such as chemoattractants, cytokines, and chemokines (Menon and Gaestel, 2018). These signals are transduced into a p38^MAPK^-mediated cellular response through receptors, generally at the cell surface and the subsequent initiation of MAPK signaling cascades, as described above. Through phosphorylation and activation of a range of targets, p38^MAPK^ co-ordinates a cellular response to these inputs, which can include the amplification of inflammatory responses though synthesis and release of pro-inflammatory cytokines (Ronkina et al., 2010; Menon and Gaestel, 2018) and altering the morphology or motility of the cell through changes in the actin cytoskeleton (Scharf et al., 2013). Many of these processes are co-ordinated by MK2.

MK2 is activated by the α and β isoforms of p38^MAPK^, through phosphorylation at Thr-222, Ser-272 and Thr-334 (Freshney et al., 1994; Han et al., 1994; Rouse et al., 1994; Li et al., 2018). MK2 has multiple targets for phosphorylation, but its primary function is as a master regulator of RNA binding proteins (Culbert et al., 2006; Gaestel, 2006; Gais et al., 2010; Gurgis et al., 2014; Soni et al., 2019). Experimental evidence indicates that MK2 regulates the stability of genes that harbor adenine/uridine-rich elements (AREs) in their 3’-untranslated region (3’-UTRs). The control of mRNA stability and translation by MK2 is dependent on AU-rich elements in the 3’ untranslated mRNA region, and on RNA-binding proteins. Deletion of MK2 leads to an impaired inflammatory response, which is mainly due to reduced *TNF*-mRNA stability or translation (Gaestel, 2006).

## Activation of p38^MAPK^-Mk2 Signaling Axis in Response to Inflammation

**FIGURE 1:**
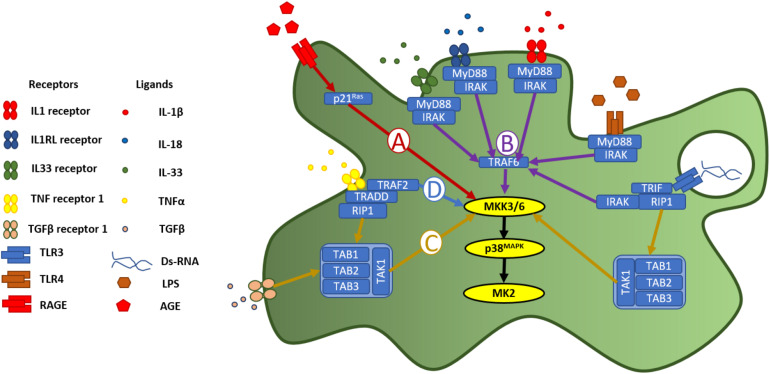
p38^MAPK^-MK2 axis is activated in response to diverse inflammatory stimuli. p38^MAPK^ is a point of convergence for a wide diversity of intracellular signaling pathways triggered by pattern recognition receptors and receptors for cytokines and other inflammatory signaling molecules. Following activation of RAGE, **(A)** the p38^MAPK^-MK2 signaling axis is activated downstream of p21^*Ras*^, via MKK3/6 (Yeh et al., 2001; Ott et al., 2014). Activation of receptors in the IL1R superfamily **(B)**, including the toll-like receptors, signal through MyD88 and IRAK, to phosphorylate TRAF6, which in turn activates MKK3/6 and then p38^MAPK^ (Martin and Wesche, 2002; Dunne and O’Neill, 2003). **(C)** Activation of TGFβR, meanwhile leads to activation of p38^MAPK^, via TAK1 phosphorylation of MKK3/6 (Yu et al., 2002). This TAK1-dependent pathway is also activated downstream of TNFR1 and TLR3 activation, via RIP1 (Shin et al., 2009). **(D)** Activation of TNF receptor 1 activates p38^MAPK^ via TRAF2 phosphorylation of MKK3/6.

The p38^MAPK^-MK2 signaling axis is embedded in a signaling cycle both downstream of receptors for inflammatory stimuli and upstream of the synthesis and release of pro-inflammatory signaling molecules, allowing it to function as an amplifier of inflammation (Menon and Gaestel, 2018). A broad variety of receptors for inflammatory stimuli and signaling molecules converging through shared pathways, unite at this signaling axis to produce a cellular response (Figure 1). The immune system uses pattern recognition receptors (PRRs) (Takeuchi and Akira, 2010; Amarante-Mendes et al., 2018) for the detection of molecules associated with pathogens (pathogen-associated molecular patterns (PAMPs) and damage (damage-associated molecular patters (DAMPs) (Tang et al., 2012). The receptor for advanced glycation end-products (RAGE) is a multi-ligand, cell-surface PRR, which initiates cellular responses to DAMPs. It binds advanced glycation end-products (AGEs)- proteins or lipids, which have become glycated after exposure to sugars—which accumulate in the extracellular space with increasing age, inflammation, oxidative stress, or as a consequence of ischemia-reperfusion or high glucose (Singh et al., 2001). As well as binding AGEs, RAGE is also activated by calgranulins (Hofmann et al., 1999), amyloid-beta (Aβ) peptides (Yan et al., 1996; Takuma et al., 2009; Piras et al., 2016), associated with Alzheimer’s disease, and high-mobility group protein 1 (HMGB1) (Huebener et al., 2015), a chromatin protein which functions in the nucleus, but is released by immune cells as a leaderless cytokine (Klune et al., 2008). Activation of RAGE initiates, via p21^*Ras*^ protein activator 1 (Figure 1A), a MAPK signaling cascade culminating in phosphorylation of MK2 by p38^MAPK^ (Ott et al., 2014). In immune cells, the p38^MAPK^-MK2 signaling axis is necessary for the synthesis and release of inflammatory cytokines in response to RAGE activation (Yeh et al., 2001).

Toll-like receptors (TLRs) are a family of archetypal PRRs, which detect molecular patterns associated with pathogens (Akira et al., 2001; Janeway and Medzhitov, 2002; Figure 1B). Lipopolysaccharide (LPS), the material from which the cell wall of gram positive bacteria is constructed, is a ligand for toll-like receptor 4 (TLR4) (Park and Lee, 2013), and can be used experimentally to induce an inflammatory response. Indeed, p38^MAPK^ was first discovered through its activation, via tyrosine phosphorylation, in response to LPS-induced TLR4 activation (Han et al., 1993, 1994). Toll-like receptor 3 (TLR3) meanwhile, is expressed in the cytoplasm or in the membrane of endosomes (Chaturvedi and Pierce, 2009) where it detects strings of nucleotides indicative of a viral infection (Perales-Linares and Navas-Martin, 2013) (Figure 1B). Polyriboiosinic:polyribocytidylic acid [poly(I:C)] is a synthetic analog of double-stranded RNA, which can be used experimentally as an immunostimulant to induce an inflammatory response via activation of TLR3 (Farkas et al., 2019). Using Poly(I:C) to stimulate cells, it was demonstrated that the cellular response to activation of TLR3 is dependent on the p38^MAPK^-MK2 signaling axis, through a pathway involving myeloid differentiation primary response 88 (myD88) and TIR-domain-containing adaptor-inducing interferon-β (TRIF) (Johnsen et al., 2012; Figure 1B). MK2, therefore functions an effector molecule in cellular responses to detection of both bacterial or viral pathogens.

The TLRs are part of a larger family of receptors, characterized by a toll-IL-1-receptor (TIR) domain (Boraschi et al., 2018). This group includes the interleukin-1 family of receptors, composed of interleukin receptor-1 (ILR1) [a receptor for the leaderless pro-inflammatory cytokines interleukin-1α and β (IL-1α and IL-1β)], interleukin receptor-18 (ILR18), interleukin receptor-like 1 (IL1RL1), receptors for interleukin-18 and interleukin-33, respectively (Dinarello, 2018). The IL-1 receptor family signal through MyD88 and interleukin-1 receptor associated kinase (IRAK) (Martin and Wesche, 2002). IRAK activates the classic MAPK-signaling pathway via tumor necrosis factor receptor associated factor 6 (TRAF6), leading, via MKK3 and MKK6 to activation of the p38^MAPK^-MK2 signaling axis (Dunne and O’Neill, 2003; Figure 1B). Evidence for MK2 phosphorylation following activation of receptors in the IL-1R family includes in responses to IL-1β (Raingeaud et al., 1995; Dunne and O’Neill, 2003), IL-33 (Helbig et al., 2020; Petrova et al., 2020), and IL-18 (Dunne and O’Neill, 2003).

Transforming growth factor-β (TGF-β) is a multifunctional cytokine, released in latent form and activated by proteolysis by matrix metalloproteinases on the cell surface (Karsdal et al., 2002). Upon activation, TGFβ functions as a ligand for dimeric, single pass serine/threonine receptors made up of the subunits TGFβ receptor 1 (TGFβR1/ALK5) and TGFβ receptor 2 (TGFβR2). Binding of TGFβ to TGFβR1 activates a signaling cascade involving MKK3/6, p38^MAPK^ and MK2, through the phosphorylation of phosphorylation of TGFβ-activated kinase 1 (TAK1) (Figure 1C; Yu et al., 2002).

Tumor necrosis factor-α (TNFα) (Menon and Gaestel, 2018) is a pro-inflammatory cytokine released into the extracellular space, largely by leukocytes during the acute stage of inflammation (Parameswaran and Patial, 2010; Wajant and Siegmund, 2019), and functions as the ligand for two cell surface receptors: tumor necrosis factor receptor 1 and 2 (TNFR1 and TNFR2) (Idriss and Naismith, 2000). TNFR1 activates MAPK-signaling cascades via TNFR1-associated death domain (TRADD) and TNF receptor-associated factor 2 (TRAF2). TRAF2 phosphorylates MKK3/6 (Shi and Sun, 2018; Figure 1D). A separate pathway leading to phosphorylation of MKK3/6 is via receptor-interacting protein-1 (RIP1) phosphorylation of TAK1 (Shin et al., 2009) TAK1 phosphorylation is a point of confluence not only with signaling cascades downstream of TGFβR1 (described above), but also via RIP1 activated by TLR3 on endosomes (Kawasaki and Kawai, 2014).

The full range of inflammatory pathways, which lead to activation of the p38^MAPK^-MK2 axis is broader than can be described within the scope of this review. Other receptors for inflammatory stimuli which have been demonstrated to activate the p38^MAPK^-MK2 signaling axis include (but are not limited to): monocyte chemoattractant protein 1/chemotactic cytokine ligand 2 (MCP-1/CCL2) activation of chemotactic cytokine receptor 2 (CCR2) (Cho and Gruol, 2008), and interferon-γ activation of interferon-γ receptor (IFNGR) (Culbert et al., 2006).

## p38^MAPK^-MK2 Signaling Axis in Neurons

**FIGURE 2:**
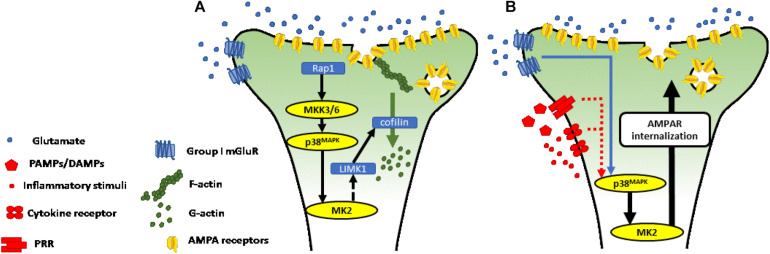
**(A)** p38^MAPK^-MK2 axis is necessary for mGluR-LTD at Schaffer collateral—CA1 synapses. Activation of perisynaptic group I mGluR induced long-term depression is dependent on activation of the p38^MAPK^-MK2 axis. The p38^MAPK^-MK2-LIMK1-cofilin signaling cascade leads to depolymerization of the actin cytoskeleton, with a decrease in ratio of filamentous (F-) to globular (G-) actin (Meng et al., 2002; Kobayashi et al., 2006; Honkura et al., 2008). **(B)** Putative mechanism through which p38^MAPK^-MK2 signaling axis mediates neurotransmission in response to inflammatory stimuli. Pattern recognition receptors, receptors for cytokines, and receptors for other inflammatory signaling molecules trigger intracellular signaling cascades, which converge at the p38^MAPK^-MK2 signaling axis (see section “Activation of p38^MAPK^-MK2 Signaling Axis in Response to Inflammation” and Figure 1 for more detail). MK2 controls cytoskeletal dynamics and receptor trafficking, as described in section “p38^MAPK^-MK2 Signaling Axis in Neurons” and **A**). Through this mechanism, we propose that the p38^MAPK^-MK2 signaling axis controls synapse strength in response to diverse inflammatory stimuli.

All p38^MAPK^ isoforms are expressed in the mouse brain, with the p38α and β isoforms particularly prominent in the cerebral cortex and hippocampus, with p38α most abundant in neurons (Lee et al., 2000; Beardmore et al., 2005). MK2 is also constitutively expressed in neurons, where it is activated by p38α and β and plays an important role in mediating synaptic plasticity induced by group I metabotropic glutamate receptors (mGluR1/5) (Kemp and Bashir, 1997; Palmer et al., 1997; Moult et al., 2008; Gladding et al., 2009; Sanderson et al., 2016; Privitera et al., 2019; Figure 2A). In the hippocampus, activation of perisynaptic mGluR5 on excitatory neurons (Lujan et al., 1996) mediates a prolonged activity-dependent reduction in the synapse efficacy (long-term depression: mGluR-LTD) (Gladding et al., 2009). The mechanisms underlying mGluR-LTD have been extensively studied at hippocampal *Schaffer collateral*-CA1 synapses, where it can be induced through the application of paired-pulse low frequency stimulation (Kemp and Bashir, 1997) or with the mGluR5 agonist (S)-3,5-dihydroxyphenylglycine (DHPG) (Palmer et al., 1997).

The p38^MAPK^-MK2 signaling axis (Thomas et al., 2008; Eales et al., 2014), activated downstream of mGluR5, controls the molecular machinery in dendritic spines necessary for induction of mGluR-LTD. This includes the molecular regulators of actin remodeling and trafficking of the α-Amino-3-hydroxy-5-methyl-4-isoxazoleproprionic acid receptor (AMPA-R) subunits, GluA1 and GluA2 (Corrêa and Eales, 2012; Eales et al., 2014; Wall et al., 2018; Privitera et al., 2019). The molecular mechanisms underlying synaptic plasticity often vary in different brain regions (Gladding et al., 2009). At CA1 dendrites, mGluR-LTD is mediated by Ca^2+^-independent mechanisms dependent on a cascade triggered by mGluR5 activation leading to the sequential activation of repressor activator protein 1 (Rap1), MKK3/6, p38^MAPK^ and MK2 (Huang et al., 2004).

The morphology of dendritic spines, and GluA1/GluA2 trafficking is controlled through proteins which act to reorganize dynamic elements of the cytoskeleton. A principal target of MK2, in this regard, is LIM domain kinase 1 (LIMK1), which is phosphorylated at Ser-323 (Kobayashi et al., 2006) and regulates the activity of proteins involved in cytoskeletal remodeling. Cofilin1 is a target of LIMK1 (Meng et al., 2002) and, when activated coordinates the depolymerization of actin filaments (F-actin) into actin monomers (G-actin). The dynamic ratio between F-actin and G-actin is largely responsible for the structural changes of the dendritic spine. A switch toward G-actin, triggered through the p38^MAPK^-MK2-LIMK-cofilin1 signaling cascade drives the spine toward decreased size and away from a mushroom-like morphology (Honkura et al., 2008; Figure 2A). These structural changes are associated with LTD and decreased neurotransmission through the synapse.

AMPA-R trafficking mediating mGluR-LTD is largely coordinated by the expression of the activity-dependent immediate early gene, Arc/Arg3.1 (Snyder et al., 2001; Bramham et al., 2008; Park et al., 2008; Waung et al., 2008; Smith-Hicks et al., 2010; Wall et al., 2018). Transcripts of Arc/Arg3.1 are rapidly transported to dendrites, enabling locally controlled translation at post-synaptic ribosomes (Bramham et al., 2008). Protein expression of Arc/Arg3.1 is regulated at individual dendritic spines through control of translational (Waung et al., 2008); polyubiquitination and targeting for proteolysis (Mabb and Ehlers, 2010; Mabb et al., 2014; Mabb and Ehlers, 2018; Wall et al., 2018). Translation and expression of Arc/Arg3.1 facilitates AMPA-R endocytosis via interaction with cytoskeletal elements (Chowdhury et al., 2006; Waung et al., 2008; DaSilva et al., 2016; Wall and Corrêa, 2018). Although the mechanism by which the MK2 cascade may control Arc/Arg3.1 expression is unclear, the Arc/Arg3.1 gene contains the serum response element (SRE), which may be directly phosphorylated by serum response factors (SRF) (Ronkina et al., 2011), under the control of MK2. Additionally, as a master regulator of RNA-binding proteins (Soni et al., 2019), MK2 may also control the phosphorylation state of proteins involved in regulating local translation and may regulate Arc/Arg3.1 expression both at the transcriptional and local translational level.

The molecular events controlled by the p38^MAPK^-MK2 signaling axis mediate changes in synaptic strength that impact at cognitive and behavioral levels. Intact mGluR-LTD is necessary for maintaining cognitive flexibility (Eales et al., 2014), memory extinction (Lüscher and Huber, 2010), and setting the ground for functional long-term potentiation (LTP) at the synapse (Walsh et al., 2002), underlying the formation of new memories. The Barnes maze task, an assay for spatial learning, can be used to measure levels of cognitive flexibility, by adjusting the position of the exit hole after a period of training. While the initial spatial learning task was intact in MK2 knockout mice, they showed severe impairments in relearning the task, demonstrating a deficit in reversal learning (Privitera et al., 2019). These deficits resonate with impairments seen in Fragile-X syndrome (Bear et al., 2004) and Alzheimer’s disease (Shankar et al., 2008), which involve dysfunction of mGluR-LTD.

## p38^MAPK^-MK2 Signaling Axis at Confluence of Inflammatory and Synaptic Signaling

Under inflammatory conditions, adaptive structural plasticity of dendritic spines is impaired (Zou et al., 2016) and AMPA-R internalization is increased (Park and Lee, 2013), though the mechanisms underlying these changes are not fully characterized. The p38^MAPK^-MK2 signaling axis, embedded both in pathways downstream of receptors for a broad range of inflammatory mediators and a necessary effector of mGluR-LTD, is a prime candidate for mediating inflammation-induced changes in neurotransmission. Activated downstream of stress and inflammatory signals in other contexts, we propose that MK2, expressed in neurons, may be an important player in the dysregulation of cognitive and effective functions following injury, infection or as a consequence of autoimmune reactions (Figure 2B).

Central to this hypothesis is the neuronal expression of receptors for cytokines and/or chemokines, PRRs and other receptors that respond to inflammatory stimuli. Indeed, many of the receptors that activate the p38^MAPK^-MK2 signaling axis in immune cells are also expressed in neurons, either constitutively, or induced under inflammatory conditions. Neurons constitutively express TNFR1 (Probert, 2015), while TNFR2 is largely restricted immune and glial cells. Neuronal TNFR1 can also be upregulated in response to insult (Pradillo et al., 2005), mediating processes involved in preconditioning. IL-1R, meanwhile, is expressed in neurons and, more specifically, in dendritic spines where it has been shown to influence synaptic plasticity (Prieto et al., 2015). Amongst other receptors in the IL-1R superfamily, the interleukin-18 receptor (IL-18R) is expressed in neurons, where it is involved in mediating neuronal responses to IL-18 (Alboni et al., 2010). Evidence for neuronal expression of interleukin-1 receptor like receptor (IL1RLR), also known as growth stimulation expressed gene 2 (ST2) is more limited. With expression in sensory neurons well characterized (Liu et al., 2016), but limited data supporting expression of this receptor in central neurons (Fairlie-Clarke et al., 2018).

Amongst the PRRs, there is strong evidence for both constitutive expression of TLR4 in neurons (Leow-Dyke et al., 2012), and upregulation of expression with aging and in pro-inflammatory conditions, such as high levels of extracellular Aβ (Calvo-Rodríguez et al., 2017). There is also strong evidence for endosomal TLR3 expression in neurons, where the receptor not only contributes to mediating cellular responses to pathogens, but also plays a role in regulation of neuronal morphology (Hung et al., 2018). Neuronal expression of RAGE is also well documented (Sasaki et al., 2001), with this receptor heavily implicated in the pathogenesis of neurodegenerative disorders, particularly Alzheimer’s disease (Cai et al., 2016), and in contributing to neurological sequelae associated with diabetes (Toth et al., 2007) and other systemic pathologies with an inflammatory element (Gasparotto et al., 2019). Receptors for TGF-β are also expressed in neurons, where they have been implicated in mediating cell fate in response to insult (König et al., 2005).

As described in section “Activation of p38^MAPK^-MK2 Signaling Axis in Response to Inflammation” above, these cytokine receptors and PRRs activate MAPK-signaling cascaded culminating in the p38^MAPK^-MK2 axis. As described in section “p38^MAPK^-MK2 Signaling Axis in Neurons,” phosphorylated MK2 regulates synaptic plasticity through transcriptional, post-transcriptional and post-translational control of a suite of effector proteins, which lead to changes in the morphology of dendritic spines and endocytosis of AMPA receptors. While this has been demonstrated in response to activation of group I metabotropic glutamate receptors (mGluR-LTD) (Eales et al., 2014), we suggest that the importance of MK2 in effector pathways downstream of cytokine receptors and PRRs make this molecule a prime candidate in translating inflammatory stimuli into neuronal responses.

## Conclusion and Future Perspectives

A key role for the p38^MAPK^-MK2 signaling axis has been identified as an effector of cellular responses to inflammatory signals and stimuli. The same signaling axis has also been shown to be a key link in the processes mediating mGluR-LTD. Neuroinflammation is a ubiquitous or near ubiquitous feature of neurological disorders and we identify the p38^MAPK^-MK2 signaling axis as a potentially important mechanistic link between neuroinflammation and synaptic dysregulation underlying cognitive impairments in neurological disorders, such as Alzheimer’s disease. MK2-mediated mGluR-LTD is necessary for maintaining cognitive flexibility, the loss of which is an early clinical sign of Alzheimer’s disease. Inhibitors of p38^MAPK^ have been trialed in a number of contexts where inflammation makes an important contribution to disease etiology (Lee and Kim, 2017). These inhibitors, however, have suffered from poor efficacy and a high burden of adverse effects (Hammaker and Firestein, 2010). Inhibitors which specifically target p38^MAPK^ mediated activation of MK2 or target the activity of MK2 itself may offer more promise, with a narrower focus. The p38^MAPK^-MK2 signaling axis is a promising target for therapeutic intervention, where inflammation contributes to dysregulation of neuronal network activity. More evidence, however, is necessary to clarifying the contribution of this signaling axis.

## Author Contributions

EB and SALC conceived the review and shared in writing the manuscript. Both authors contributed to the article and approved the submitted version.

## Conflict of Interest

The authors declare that the research was conducted in the absence of any commercial or financial relationships that could be construed as a potential conflict of interest.
